# Case Report: Everolimus reduced bone turnover markers but showed no clinical benefit in a patient with severe progressive osseous heteroplasia

**DOI:** 10.3389/fped.2022.936780

**Published:** 2022-11-22

**Authors:** M. Cebey-López, M. J. Currás-Tuala, J. Gómez-Rial, I. Rivero-Calle, J. Pardo-Seco, R. Mendez-Gallart, S. Pischedda, A. Gómez-Carballa, R. Barral-Arca, A. Justicia-Grande, S. Viz-Lasheras, C. Rodríguez-Tenreiro, R. Gómez, A. Salas, F. Martinón-Torres

**Affiliations:** ^1^ Genetics, Vaccines, Infectious Diseases and Pediatrics Research Group (GENVIP), Health Research Institute of Santiago de Compostela (IDIS), Santiago de Compostela, Spain; ^2^ Translational Pediatrics and Infectious Diseases, Department of Pediatrics, Hospital Clínico Universitario de Santiago de Compostela, Santiago de Compostela, Spain; ^3^ Servicio de inmunologia, Servicio de Análisis Clínicos. Hospital Clínico Universitario (SERGAS), Santiago de Compostela, Spain; ^4^ Pediatric Surgery, Hospital Clínico Universitario de Santiago, Santiago de Compostela, Spain; ^5^ Musculoskeletal Pathology Group, Institute IDIS, Santiago University Clinical Hospital (SERGAS), Santiago de Compostela, Spain; ^6^ Unidade de Xenética, Instituto de Ciencias Forenses (INCIFOR), Facultade de Medicina, Universidade de Santiago de Compostela, Santiago de Compostela, Spain; ^7^ GenPoB Research Group, Instituto de Investigación Sanitaria, Hospital Clínico Universitario de Santiago (SERGAS), Santiago de Compostela, Spain

**Keywords:** progressive osseous heteroplasia, Everolimus, bone turnover, bone metabolism, mTOR

## Abstract

**Background:**

Progressive osseous heteroplasia (POH) is an ultrarare genetic disorder characterized by an inactivating mutation in the *GNAS* gene that causes heterotopic ossification. Inhibition of the mammalian target of the rapamycin (mTOR) signalling pathway has been proposed as a therapy for progressive bone fibrodysplasia and non-genetic forms of bone heteroplasia. Herein, we describe the impact of using Everolimus as a rescue therapy for an identical twin girl exhibiting an aggressive clinical phenotype of POH.

**Methods:**

Clinical evaluation of the progression of the disease during Everolimus treatment was performed periodically. Cytokine markers involved in bone metabolism and protein markers related to bone activity were analyzed to explore bone turnover activity.

**Results:**

The patient received Everolimus therapy for 36 weeks. During treatment, no clinical improvement of the disease was perceived. Analysis of biochemical parameters, namely, β-CTX (*r*^2^ = −0.576, *P*-value = 0.016) and PNIP (*r*^2^ = −0.598, *P*-value = 0.011), indicated that bone turnover activity was significantly reduced. Additionally, bone metabolism-related biomarkers showed only a significant positive correlation with PTH levels.

**Conclusions:**

Everolimus treatment did not modify the clinical progression of the disease in an aggressive form of POH, although an impact on the protein markers studied was observed.

## Introduction

Progressive osseous heteroplasia (POH) is an ultrarare genetic condition that manifests as progressive extra-skeletal bone formation, usually in early life (1). It is caused by loss of heterozygosity at the *GNAS* locus, resulting in homozygosity, hemizygosity, or compound heterozygosity (2, 3), specifically in the G-alpha subunit, a G protein coupled to membrane receptors that are involved in controlling multiple signalling transduction pathways and several biological functions, including bone growth (osteogenesis).

POH is clinically characterized by a progressive heterotopic bone formation within the dermis and subcutaneous adipose tissue that progresses to deep connective tissue, including fascia, skeletal muscle, tendon, and ligament (4, 5). Over time, POH dermal maculopapular lesions can eventually coalesce into bone plaques that spread into deeper connective tissue, resulting in extensive ossification and ankylosis of affected joints. POH usually evolves into severe disability, limb movement limitation, and bone pain (1, 6).

Currently, no treatment has been demonstrated to resolve pre-existing bone lesions in POH (4), and, in addition, surgical resection of the affected tissues can lead to recurrences or complications (1, 7).

The mammalian target of the rapamycin (mTOR) pathway affects energy levels, nutrient availability, growth factor functions, and cellular stress, integrating both intracellular and extracellular signals (8–10). The role of mTOR complex 1 (mTORC1) is to activate the translation of proteins and regulate numerous cellular processes related to growth and differentiation. mTORC1 is also involved in postnatal bone formation and remodelling, stimulating differentiation of recruited mesenchymal stem cells (MSCs) into mature osteoblasts when it is activated and reducing osteogenesis when it is inhibited (11). Previous studies indicated that drugs targeting mTOR could slow down the symptoms of the POH disease (12–17).

Inhibition of the mTOR signalling pathway using rapamycin has proven to prevent trauma-induced heterotopic ossification through the arrest of osteogenic differentiation at the early osteoblast stage (12) and has been proposed as a therapy for progressive bone fibrodysplasia and non-genetic forms of bone heteroplasia (13, 17). In fact, rapamycin has been shown to successfully prevent trauma-induced heterotopic ossifications in animal models (13, 16, 17). Everolimus, an immunosuppressive drug, is another inhibitor of the mTORC1 complex, which performs its function through a high-affinity interaction with protein receptor FKBP12 (18).

We describe our experience with Everolimus as compassionate therapy for a twin girl suffering from a severe and rapid POH form, unresponsive to any previous treatment. Several biochemical and bone turnover- and metabolism-related biomarkers in serum during Everolimus treatment have been studied to clinically and biochemically characterize the patient’s status and eventual response to therapy.

## Materials and methods

### Clinical case description

Two identical twins aged 7 years with confirmed genetic diagnosis of POH are clinically followed in our hospital. A detailed description of the clinical case is given by Justicia-Grande et al. (19). Briefly, one of the sisters (referred to as Twin#1) shows an aggressive and disabling course of the disease, needing surgical excision to remove osseous plaques distributed throughout the body and requiring orthopaedic help to walk. The other twin sister (Twin#2) shows a practically asymptomatic clinical course of the disorder, with only mild manifestations of bone spikes. Bone biomarkers, analyzed in serum samples of both twins, revealed in Twin#1 an altered level of bone growth factor (IGF-1), lower than the normal range, and an elevated level of bone formation markers BAP, PINP, and OC. Markers related to bone reabsorption (ß-CTX), bone mineralization (calcium, phosphorus, and vitamin D), thyroid function (PTH and TSH), and autoimmune processes (autoantibodies) remained within the normal value range. In Twin#2, all the markers analyzed remained within the normal range. Any other clinical features related to PHP and PPHP were not observed in the patients. Both twins share the same de novo pathogenic mutation in the *GNAS* locus (n565–568delGACT, exon 7), detected in the blood through Sanger sequencing (20). The mutation was absent in both parents. As a consequence of the disabling progression of the illness, experimental treatment with Everolimus was only considered and approved to be administered to Twin#1; however, if there had been positive results and clear benefits, Twin#2 could have received the same treatment and therefore act as a direct clinical and analytical comparator.

Serum samples from Twin#1 were obtained during the treatment with Everolimus in order to perform a complete follow-up. Fasting samples were collected in the early morning (08:30).

The study was reviewed and approved by the Ethics Committee of Clinical Investigation of Galicia (CEIC ref. 2019/325). The parents of the patients provided their written informed consent to participate in this study.

Additionally, as the Everolimus was administered for compassionate use, all the permissions needed were obtained.

Twin#1 was treated with Everolimus for 254 days (36 weeks) and was taken with meals (14:00). Its administration was continued while it was clinically appropriate in the assessment of disease progression; it had no inadequate therapeutic effects nor presence of unacceptable side-effects. The long-term efficacy of Everolimus was evaluated through an exhaustive clinical inspection, evaluating the existence of ectopic bone attenuation or newly formed lesions in the context of POH. The drug was administered orally once a day with a charge dose of 4.5 mg/m^2^ and a maintenance dose according to target valley levels (5–9 ng/ml), adjusting the dose to reach optimal concentrations in blood. Levels of Everolimus were periodically monitored using the ADVIA Centaur CP automated immunoassays (Siemens Healthcare, Erlangen, Germany). Prophylactic therapy with cotrimoxazole was prescribed initially when Everolimus was started due to the potential immunosuppressive side effect of the drug; however, it was stopped at day 33 when it was proven no longer necessary.

Complete clinical and biochemical monitoring of the disease was performed during the treatment period administration.

### Bone turnover markers

We analysed changes in bone turnover markers (BTMs) levels at different time points since the beginning of the therapy: 2, 4, 9, 16, 30, 44, 93, 100, 114, 143,199, 212, 233, and 254 days after the onset of the treatment.

BTMs are released by osteoblasts or osteoclasts during bone remodelling. These markers are proteins or protein derivatives that respond rapidly to changes in bone physiology. BTMs were measured in the serum, namely, alkaline phosphatase (ALP), C-terminal telopeptide type 1 collagen or beta crosslaps (β-CTX), and N-terminal propeptide type I procollagen (PNIP). All these parameters were determined using the ADVIA Centaur automated immunoassays, generally used for clinical diagnostics (Siemens Healthcare, Erlangen, Germany). This is a chemolumiscence immunoassay that has a highly specific affinity in the detection of specific antibodies.

### Bone metabolism-related biomarkers

Bone metabolism-related biomarkers were analysed in the same time points of BTMs when the serum sample was available. Characterization of bone metabolism during Everolimus treatment was carried out by measuring 13 bone serum biomarkers using MILLIPLEX MAP magnetic bead immunoassay kits (Cat# HBNMAG-51K Millipore) based on Luminex xMAP technology. This assay included the following biomarkers: adrenocorticotropic hormone (ACTHA), dickkopf-1 (DKK-1), growth factor 23 (FGF-23), IL-1β, IL-6, insulin, leptin, osteocalcin (OC), osteopontin (OPN), osteoprotegerin (OPG), parathyroid hormone (PTH; also known as teriparatide), sclerostin (SOST), and tumor necrosis factor α (TNF-α). Measurement of ACTH and FGF-23 failed and was therefore eliminated from the analyses. Cytokines measurement was performed using 25 μl of serum from the patient in the treatment timeline followed by the incubation of samples with 25 μl of magnetic bead conjugates with capture antibodies in a 96-well plate overnight at 4 °C. After incubation the plate was washed, 50 μl of biotinylated detection antibody cocktail was added, and the plate was incubated at room temperature for 1 h. Then 25 μl of streptavidin-phycoerythrin was added and incubated for 30 min. After another washing step, the beads were resuspended in 100 μl buffer to read fluorescent levels on a Labscan 200 flow cytometer. Data obtained from fluorescent levels were normalized using a standard curve.

### Statistical analysis

A correlation analysis was carried out using Pearson's coefficient to evaluate the evolution of different biomarkers during Everolimus treatment. The nominal significance level was set to 0.05. Bonferroni correction was used to adjust for multiple tests. Statistical analyses were carried out using the statistical software R (http://www.r-project.org).

## Results

### Effectiveness and side effects of Everolimus

**Figure 1:**
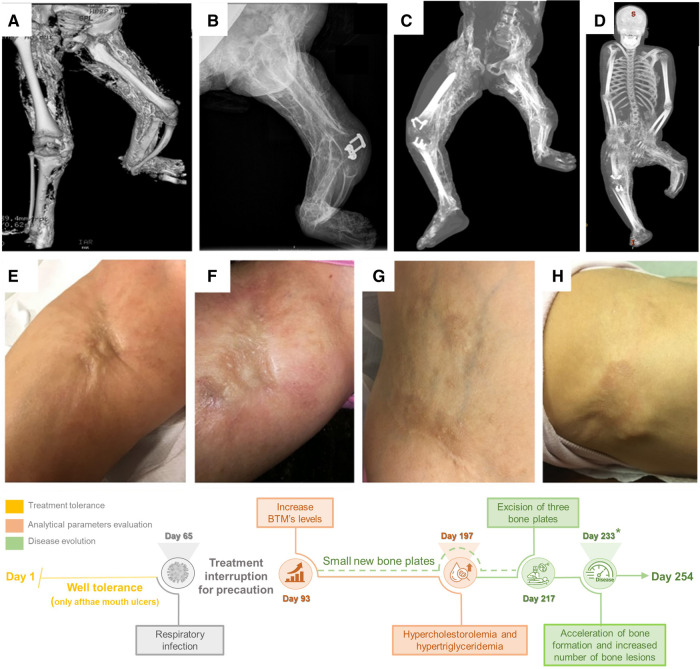
Clinical evolution of the patient with severe POH form and timeline related to Everolimus treatment (bottom). (**A–D**) Tridimensional body TC of Twin#1, showing the evolution of the disease in a 4-year period; (**E, F**) Disease progression before and after aggravation occurring around day 233* in the right groin; (**G–H**): New ectopic calcifications in the abdomen and left scapula.

A timeline resuming the relevant clinical and biochemical data of Twin#1 during Everolimus treatments is shown in Figure 1, together with images illustrating the progression of the disease in the patient. Overall, Everolimus treatment was well tolerated by the patient, with only aphthae-mouth ulcers and difficulting swallowing reported, which were only present when high therapy levels were reached and rapidly disappeared when drug levels were adjusted. No immunosuppression, non-infectious pneumonitis, hypersensitivity or renal insufficiency was experienced during the treatment despite being described as potential side effects of Everolimus according to the data sheet. However, treatment was decided to be interrupted for 28 days (from day 65 to 93) for precaution due to a viral respiratory infection.

The clinical progression of the disease was overall stable during the treatment course; thus, in this period, only a minor rising of the existing plates and onset of small new osseous plates could be observed by clinicians. On day 217, a programmed exeresis of three bone plates that already existed before Everolimus treatment was carried out. On day 233, a significant clinical acceleration of bone formation and an increased number of bone lesions were noted (Figure 1).

Analytical routine parameters were evaluated at different time points during treatment (Supplementary Table S1). Hypercholesterolemia and hypertriglyceridemia were detected on day 197 (20/9/2018). We found that glucose (*r*^2^ = 0.720, *P*-value = 0.001) was significantly correlated with Everolimus blood concentration levels. Albumin (*r*^2^ = 0.555, *P*-value = 0.021) and uric acid (*r*^2^ = −0.502, *P*-value = 0.040) were also significantly correlated, although these values did not surpass the Bonferroni correction.

### Biochemical and bone biomarkers monitoring during immunosuppressive treatment

**Figure 2:**
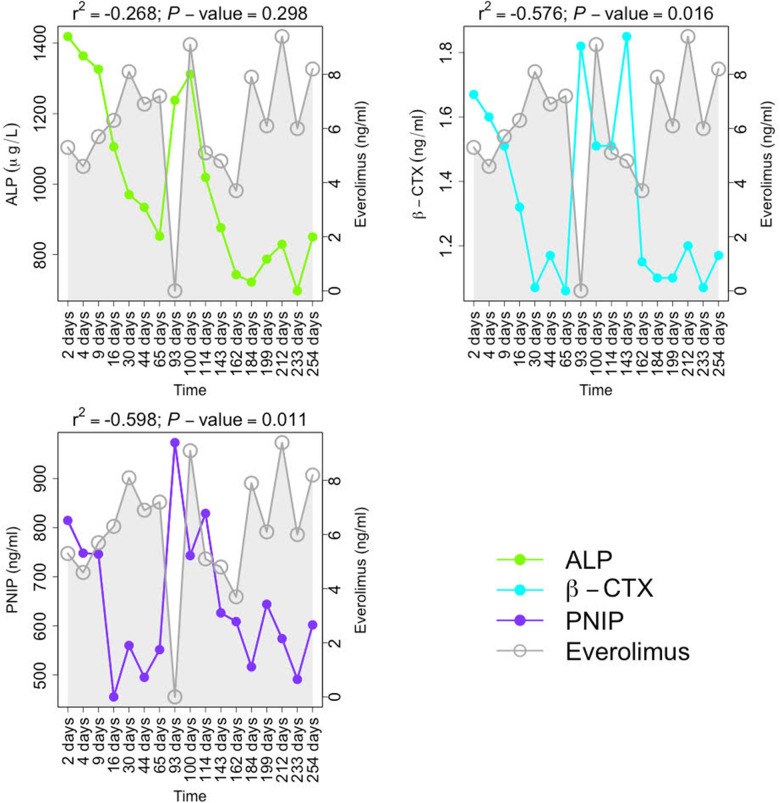
Follow-up of bone turnover activity biomarkers (BTMs) during immunosuppressive treatment with Everolimus. ALP is expressed in μg/L; β-crosslap, PNIP, and Everolimus levels are measured in ng/ml. Pearson's correlation coefficients between the corresponding BTM and Everolimus levels are displayed with the *P*-values.

Bone turnover and bone-related biomarkers were monitored during Everolimus treatment. Follow-up of these BTMs (ALP, β-CTX, and PNIP) during the treatment is shown in Figure 2. All of these serum BTMs showed a negative correlation with Everolimus serum levels, so when levels of Everolimus increased all bone variables decreased and vice versa, a short period of Everolimus decreased at time point at 93 days (Figure 1), followed a clear increased in bone variables. In this sense, β-CTX (*r*^2^ = −0.576, *P*-value = 0.016) and PNIP were significantly correlated (*r*^2^ = −0.598, *P*-value = 0.011), but on the other hand, ALP correlation was not statistically significant (*r*^2^ = −0.268, *P*-value = 0.298).

**Figure 3:**
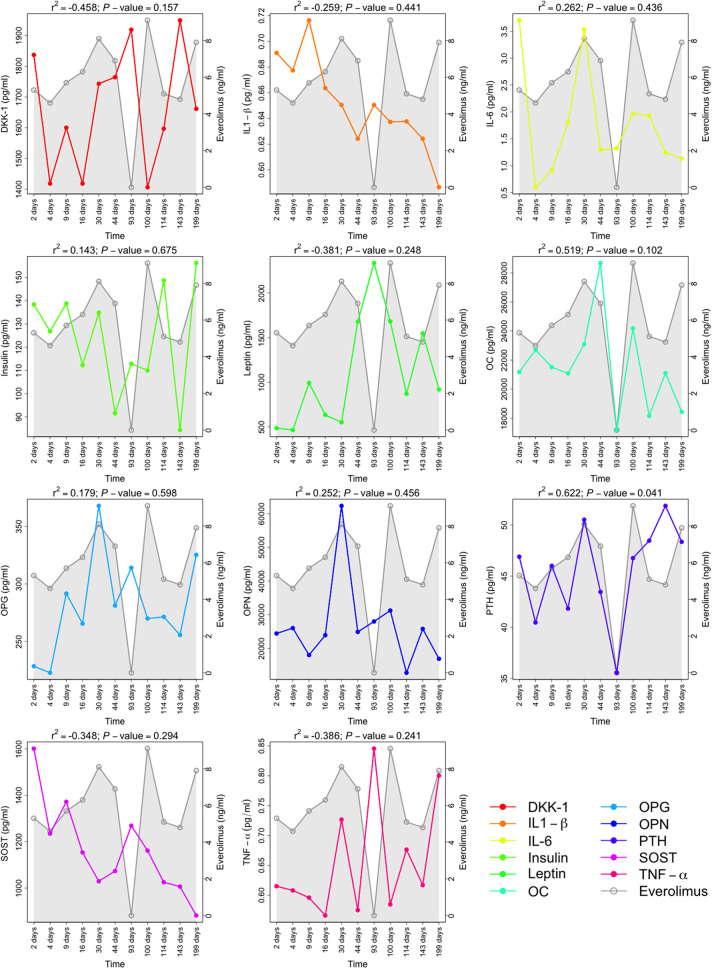
Follow-up of the cytokines as bone metabolism-related biomarkers during immunosuppressive treatment with Everolimus. All the biomarkers levels presented are expressed in pg/ml, and Everolimus serum levels are measured in ng/ml. Pearson's correlation coefficient between the corresponding bone metabolism-related markers and Everolimus levels are displayed with the *P*-values. The bone metabolism-related markers presented are DKK-1, Dickkopf-related protein 1; IL1-β, interleukin 1 beta); L-6, interleukin 6; OC, osteocalcine; OPG, osteoprotegerin; OPN, osteopontin; PTH, parathyroid hormone; SOST, sclerostin; and TNF-α, tumor necrosis factor-alpha.

In contrast to BTMs, from the bone metabolism-related biomarkers, only PTH levels showed a significant positive correlation with Everolimus serum levels (*r*^2^ = 0.622, *P*-value = 0.041) (Figure 3). We did not find a significant negative correlation between Everolimus treatment and the two inhibitors of the wingless integrated pathway (Wnt), namely, DKK1 and SOST (*r*^2^ = −0.458, *P*-value = 0.157; *r*^2^ = −0.348, *P*-value = 0.294; respectively). OC (*r*^2^ = 0.519, *P*-value = 0.102), OPG (*r*^2^ = 0.179, *P*-value = 0.598), and OPN (*r*^2^ = 0.252, *P*-value = 0.456) levels showed a positive correlation but again with significance level below the nominal threshold.

## Discussion

The two discordant monochorionic twins described in the present study represent a unique case worldwide of POH: while one of the sisters presents a severe clinical course of the disease (Twin#1), the other evolves almost asymptomatically (Twin#2) (19). The discordance observed in the disease manifestations could reflect a possible phenomenon of superimposted mosaicism, as described recently by Happle (21, 22). According to this theory, an early postzygotic event of loss of heterozygosity in the *GNAS* gene could have occurred in the most affected twin, causing a possible loss of the corresponding wild-type allele, and as a consequence, a homozygous or hemizygous cell.

Due to the rapid progression of the disease in Twin#1, several treatments were administrated. Previous failed treatment attempts by order of administration were (1) mecarsemin (rhIGF-1): dose: 0.04 mg/day, length of treatment: 15 days, no adverse events detected but worsened serum markers with same clinical manifestations; (2) naproxen: dose 100 mg/day, length of treatment: 40 days, adverse events: severe aphtous ulcers which were the cause of discontinuation; (3) topical tretinoin: dose: 0.10% and 0.025%, length of treatment: 10 days, adverse events: red, swollen rash in the chosen regions, cause of discontinuation: ossification over the scapula grew; (4) oral retinoid (acitretin): dose: 10 mg/day, length of treatment: 90 days, no adverse events, but she presented with coalescence of bony spikes of the back and progression of the plate over the left scapula, as well as appearance of new spikes surrounding the abdominal plates, which was the cause of discontinuation; (5) pamidronate: dose: 2.5 mg/kg, length of treatment: 3 days, adverse events: worsened myalgia and asthenia and onset of low-grade fever, manifestations of POH progressed, which was the cause of discontinuation; (6) itraconazole: dose: 6.6 mg/kg/q.d during 90 days followed by dose: 9.5 mg/kg/q.d during 30 days, no adverse events but biochemical markers of bone formation returned to previous levels, and there was an absence of clinical improvement in the disease progression which was the cause of discontinuation; (7) methylprednisolone: dose: 20 mg/kg/q.d during 5 days followed by slow tapering during 180 days, no adverse events but there was an absence of clinical improvement in the disease progression, despite the reduction of markers of bone formation after the initial bolus, which was the cause of discontinuation; and (8) indomethacin: dose: 3–4 mg/kg/b.i.d, during 180 days, no adverse events but there was an absence of clinical improvement in the disease progression, which was the cause of discontinuation.

To date, our experience has been unsuccessful in altering the course of the disease. Given the severity and rapid progression of the clinical course of POH, as well as the lack of response to any previous therapeutic attempt in Twin#1, Everolimus therapy was considered. The rationale for Everolimus use was based on its known inhibitory effect on the formation of heterotopic ossifications (12–17). Exhaustive clinical and biochemical follow-up during treatment was carried out. In order to evaluate the effect of this therapy, we analyzed different serum markers related to bone metabolism and turnover.

Fragments from type I collagen, the most abundant protein in bone, are used to determine bone anabolic and catabolic activity. β-CTX is a telopeptide released from type I collagen during osteoclastic resorption of bone, and its level in blood reflects the bone resorption activity. On the other hand, PNIP is a marker of collagen secretion by osteoblasts and reflects anabolic activity (23).

BTMs negatively correlated with Everolimus levels (Figure 2**)**, with a statistically significant correlation in the variables β-CTX and PNIP. In agreement with this observation, previous studies described that Everolimus was able to reduce bone turnover activity via the mammalian target of rapamycin (mTOR) inhibition (24–27).

When the correlation of Everolimus treatment was evaluated with other bone metabolism-related markers (Figure 3), only changes in PTH reached statistical significance and correlated positively with Everolimus treatment. PTH, the hormone secreted by parathyroid glands, has been strongly associated with bone regulation. It is characterized by a paradoxical behaviour: It promotes bone resorption to release ionic calcium to the blood (28) when administered continuously, and it has a strong net anabolic effect that causes bone formation when administered intermittently and at a low dose (29). In fact, nowadays PTH is used as a unique bone-forming drug for osteoporosis treatment (30). Remarkably, despite the described inhibitory effect of Everolimus on the formation of heterotopic ossifications, PTH serum levels showed a positive correlation with Everolimus treatment. However, calcium levels remained unchanged during the follow-up treatment. This observation could be consistent with a mutation in *GNAS*. Thus, genetic mutations affecting the Gs-alpha, are responsible for several human diseases for which the clinical findings result, in some of them, is abnormal PTH signaling (31). Nonetheless, previous studies have described that POH patients have functional PTH receptor signalling (32). This fact was also observed in both twins.

Among the different bone anabolic pathways, the activation of the Wnt pathway was noticeable (33), and overactivation of this pathway has been associated with osteopetrosis (34). Thus, Wnt/*β*-catenin signalling inhibition has been proposed as a potential treatment for heterotopic ossification (35). Moreover, it is well-known that some PTH anabolic effects are mediated by the activation of the Wnt pathway (33). In our results, serum levels of DKK-1 and SOST (sclerostin), two inhibitors of the Wnt pathway (36–38), showed a negative (but not statistically significant) correlation with Everolimus treatment.

During treatment, correlation of the glucose levels was observed with Everolimus serum concentrations. mTOR is a kinase found in two distinct protein complexes: mTORC1, implicated in the regulation of several cellular processes related to growth and differentiation, and mTORC2, which participates in the insulin signaling cascade. It was previously described by Lamming et al. (39) that rapamycin, another inhibitor of the mTOR, also binds to the FKBP12 and directly inhibits mTORC1. Secondarily, chronic exposure of rapamycin inhibits mTORC2 assembly, which may be associated with metabolic complications, including glucose intolerance. Abnormal lipid profiles were also described with inhibitors of mTOR. mTORC1 regulates lipid synthesis mainly through sterol-regulatory-element-binding protein transcription factors (SREBP1), while this mechanism is not completely understood yet (40).

The main limitations of this study were the lack of data on some bone biomarker time-points, that did not allow to have a global view of the trend of these biomarkers throughout the administration period of everolimus. On the other hand, we have observed that POH is a tissue-specific disease, this means that the parameters measured in the blood do not fully represent the whole progression of the disease. Also, the singular features of the present POH case are, to the best of our knowledge, absolutely unique in the literature, and therefore there is no way to validate the current findings on independent clinical cases.

## Conclusions

Overall, our results indicate that compassionate use of Everolimus treatment for a severe form of POH was adequately tolerated and had a moderate impact on the osseous turnover biomarker pattern. A nonsignificant increase of osteocalcin, the main pro-osteoblastic factor, was observed; this finding could suggest an attempt of the bone tissue to recover the high osteoblastic activity. Nevertheless, empirical rescue treatment with Everolimus did not manage to modify the clinical course of the disease, and therefore, treatment was finally discontinued.

## Data Availability

The raw data supporting the conclusions of this article will be made available by the authors without undue reservation.

## Supplementary material

Supplementary Table 1 Correlation between haematological and biochemical parameters and Everolimus serum concentrations.
