# Supplementary material for: Case Report: Everolimus reduced bone turnover markers but showed no clinical benefit in a patient with severe progressive osseous heteroplasia
Source: Front Pediatr. 2022 Nov 22;10:936780. doi: 10.3389/fped.2022.936780 (PMC9723155; doi:10.3389/fped.2022.936780)
Supplement: Supplementary Table 1 — Correlation between haematological and biochemical parameters and Everolimus serum concentrations. [file Table1.docx]

| **Variable** | **Pearson's correlation coefficient (*P*-value)** |
| --- | --- |
| Leukocytes | -0.218 (0.400) |
| Red blood cells | 0.441 (0.077) |
| Hemoglobin | 0.188 (0.470) |
| Hematocrit | 0.144 (0.580) |
| MCV | -0.155 (0.554) |
| MCH | -0.132 (0.615) |
| MCHC | 0.106 (0.686) |
| DUH | 0.195 (0.453) |
| Platelets | -0.323 (0.205) |
| MPV | -0.165 (0.526) |
| Microcytics | 0.046 (0.860) |
| Hypochromics | 0.007 (0.980) |
| Neuthophils | -0.333 (0.191) |
| Lymphocytes | 0.226 (0.383) |
| Monocytes | -0.113 (0.665) |
| Eosinophils | 0.223 (0.389) |
| Basophils | 0.197 (0.448) |
| Large unstained cells | -0.174 (0.504) |
| Erythrocyte sedimentation rate | 0.400 (0.112) |
| Glucose | 0.720 (0.001) |
| Urea | 0.084 (0.748) |
| Uric acid | -0.502 (0.04) |
| Creatinine | 0.367 (0.148) |
| Sodium | 0.187 (0.473) |
| Potassium | 0.029 (0.913) |
| Total bilirubin | -0.133 (0.624) |
| Aspartate aminotransferase | 0.006 (0.980) |
| Alanine transaminase | 0.095 (0.716) |
| Gamma-glutamyltransferase | 0.038 (0.915) |
| Total proteins | 0.406 (0.106) |
| Albumin | 0.555 (0.021) |
| Calcium | 0.450 (0.081) |
| Cholesterol | 0.432 (0.095) |
| Triglycerides | 0.418 (0.095) |

**Table S1**
